# Crystal structure of ethyl 2-cyano-2-(1,3-di­thian-2-yl­idene)acetate

**DOI:** 10.1107/S2056989017017893

**Published:** 2018-01-01

**Authors:** Wafia Boukhedena, Abdelali Fiala, Hayet Brahim Ladouani, Salah Eddine Lemallem, Noudjoud Hamdouni, Ali Boudjada

**Affiliations:** a Unité de Recherche de Chimie de l’Environnement et Moléculaire Σtructurale CHEMS, Université des Frères Mentouri Constantine, Constantine, Algeria; b Laboratoire de Cristallographie, Département de Physique, Université Mentouri-Constantine, 25000 Constantine, Algeria

**Keywords:** crystal structure, 1,3-di­thian-2-yl­idene, twist-boat conformation

## Abstract

The title compound, C_9_H_11_NO_2_S_2_, contains a 1,3-di­thiane ring which has a twist-boat conformation. The dihedral angle between the mean planes of the ethyl acetate group and the di­thiane ring is 17.56 (13)°. In the crystal, mol­ecules stack in layers up the *a*-axis direction, however, there are no significant inter­molecular inter­actions present.

## Abstract 2

In the title compound, ethyl 2-cyano-2-(1,3-di­thian-2-yl­idene)acetate, the six-membered 1,3-di­thiane ring has a twist-boat conformation. In the crystal, the mol­ecule stack in layers up the *a* axis; there are no significant inter­molecular inter­actions present.

## Chemical context   

The derivatives of compounds such as α-oxo-ketene di­thio­acetals may undergo various transformations, in addition to the reactions involving the carbonyl group, C=C double bond, or the sulfur atoms. The emphasis in recent years has focused on the development of new and efficient inter­mediates. Some examples include (*a*) the preparation of highly regioselective compounds in a one-step reaction [the first example to be reported was the regiospecific synthesis of poly-substituted phenols from 1,5-dielectrophiles, *via* the five carbon atoms that are available in the structures of acenoyl ketene di­thio­acetals (Bi *et al.*, 2005 ▸)]; (*b*) the synthesis of complex mol­ecules based on new efficient and cost-effective reactions because they allow more than one transformation into a single synthetic sequence (Dömling *et al.*, 2012 ▸; Tietze *et al.*, 2006 ▸); (*c*) the preparation of tri­fluoro­methyl-containing organic compounds of particular inter­est in the pharmaceutical and agrochemical fields due to their lipophilicity, hydro­phobic properties and stable metabolic character (Furuya *et al.*, 2011 ▸). Muzard and co-workers have been involved in the chemistry of tri­fluoro­methyl­ketene di­thio­acetals, especially perfluoro­ketene di­thio­acetals, and have reported in their work the preparation of tri­fluoro­methyl­ketene di­thio­acetals (Muzard & Portella, 1993 ▸).

The functionalization of ketene di­thio­acetals provides more powerful tools for the development of new inter­mediates (Wang *et al.*, 2011 ▸; Gao *et al.*, 2010 ▸; Hu *et al.*, 2012 ▸). Of such constructions on the skeleton of the ketene di­thio­acetals, especially those involving the formation of the C—C bonds using carboelectrophiles such as aldehydes, have provided an effective link between these compounds and a variety of organic compounds with other functional groups. Minami *et al.* (1996 ▸) reported in their work the synthesis of α-hy­droxy­phosphono­ketene di­thio­acetals from aldehydes. In addition, Kouno *et al.* (1998 ▸) have shown that phospho­rus enyne-containing groups and di­thiol­anes could be prepared by cross-coupling of di­thio­acetal cyclic α-(iodo­propane) with the corresponding alkyne phosphono­ketene.

The direct formation of the C—C bond has been carried out by reacting α-cyano ketene di­thio­acetal and Morita–Baylis–Hillman (MBH) alcohols resulting from the reaction of acrylo­nitrile and aryl aldehydes. This reaction led to the corresponding 1,4-penta­diene deriv­atives (Zhao *et al.*, 2007 ▸).

New synthetic pathways of various inter­mediates characterized by several functional groups have been created by transforming the α-acetyl­cetaldi­thio­acetal functional group into α-hy­droxy, α-chloro and α-bromo (Liu *et al.*, 2003 ▸) and α-ethynyl ketene (Dong *et al.*, 2005 ▸). The creation of new pathways to access such multi-functionalized compounds has also been achieved by reactions involving cleavage of the C—S bond (Dong *et al.*, 2011 ▸). It should be noted here that the functionalization of the alkyl­thio group of these compounds has led to products useful in a wide range of applications (Mahata *et al.*, 2003 ▸)

Fiala *et al.* (2007 ▸) have studied the inhibitive action of some synthesized ketene di­thio­acetal derivatives towards the corrosion of copper in aerated nitric acid solutions. They concluded that these compounds are good inhibitors of copper corrosion in this medium. The inhibitory properties of the title compound with respect to the corrosion of a transition metal in an acid medium were investigated in a separate study.

Herein, we report on the synthesis and crystal structure of ethyl 2-cyano-2-(1,3-di­thian-2-yl­idene)acetate (I). We also examined the effect of the substitution of the methyl group of methyl 2-cyano-2-(1,3-di­thian-2-yl­idene)acetate (II) (Ham­douni *et al.*, 2017 ▸) by the ethyl group of the title compound.
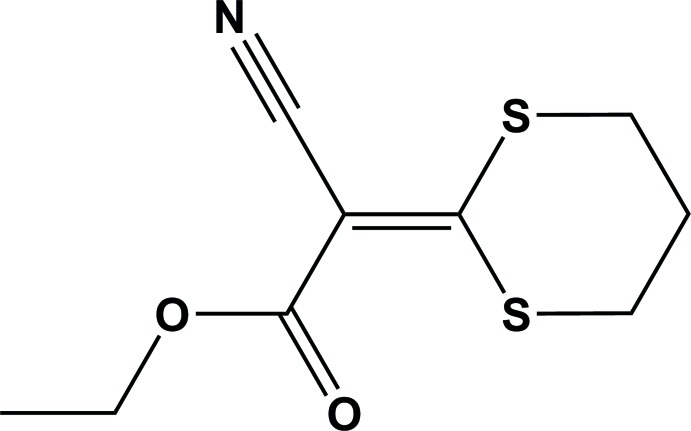


## Structural commentary   

**Figure 1:**
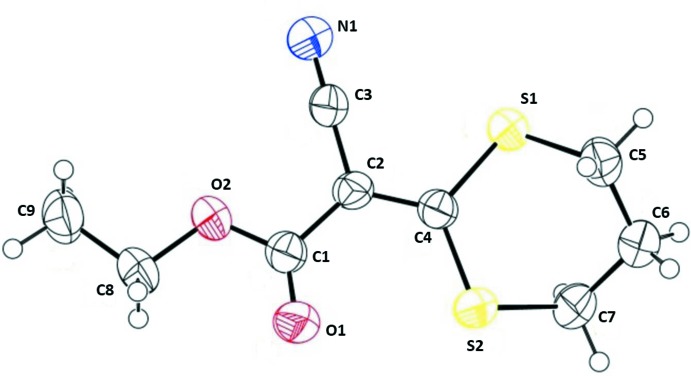
The mol­ecular structure of the title compound (I), with the atom labelling. Displacement ellipsoids are drawn at the 50% probability level.

The mol­ecular structure of the title compound (I), is illus­trated in Fig. 1 ▸. The mean planes of the ethyl acetate group [C1/C2/O1/O2/C8/C9; maximum deviation of 0.051 (2) Å for atom O2] and the dithi­azane ring (S1/S2/C1–C4) are inclined to one another by 17.56 (13)°. The di­thiane ring (S1/S2/C4–C7) has a twist-boat conformation [puckering parameters: amplitude (*Q*) = 0.909 (2) Å, θ = 89.88 (19)°, and φ = 331.65 (16)°].

The C—S bond lengths differ as expected, with the C*sp* ^2^—S bonds [S1—C4 = 1.747 (2) and S2—C4 = 1.736 (2) Å] being shorter that the C*sp* ^3^—S bonds [S1—C5 = 1.805 (3) and S2—C7 = 1.817 (3) Å]. The C2=C4 bond length is 1.378 (3) Å. All the bond lengths and angles agree well with those reported for similar compounds, for example in methyl 2-cyano-2-(1,3-di­thian-2-yl­idene)acetate, compound (II) mentioned above.

## Supra­molecular features   

**Figure 2:**
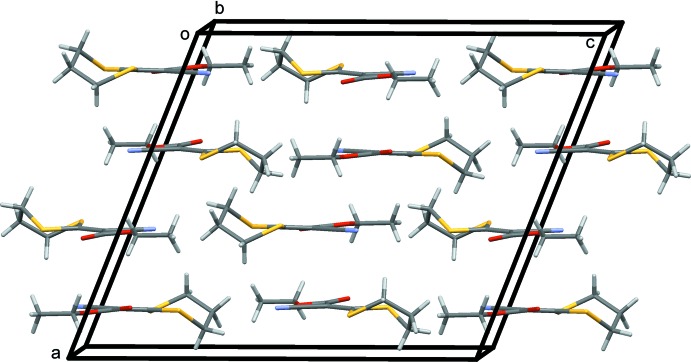
A view along the *b* axis of the crystal packing of the title compound (I).

In the crystal of (I), mol­ecules stack in layers up the *a*-axis direction (Fig. 2 ▸); however, there are no significant inter­molecular inter­actions present.

## Database survey   

**Figure 3:**
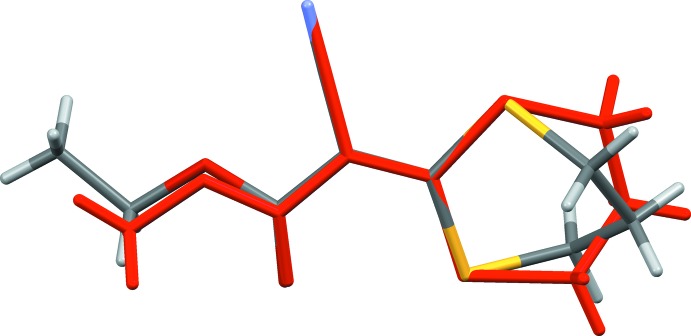
Structural overlap of compounds (I) and (II); the latter is shown in red.

A search of the Cambridge Structural Database (Version 5.38, update May 2017; Groom *et al.*, 2016 ▸) for the 2-(1,3-di­thian-2-yl­idene) skeleton yielded eight hits. They include a number of 1,2-bis­(di­thian-2-ylidenes), such as dimethyl 1,2-bis­(di­thian-2-yl­idene)-ethane-1,2-di­carboxyl­ate (ZIGVOA; Benati *et al.*, 1995 ▸). Since that update, the structure of the methyl analogue, (II), of the title compound has been reported by our group (Hamdouni *et al.*, 2017 ▸). The two structures differ essentially in the orientation of the twist-boat dithi­azane ring, as shown by the structural overlap of the two mol­ecules in Fig. 3 ▸. The puckering parameters for (I) are *Q* = 0.909 (2) Å, θ = 89.88 (19)° and φ = 331.65 (16)°, while those for (II) are *Q* = 0.632 (3) Å, θ = 106.5 (3)° and φ= 114.3 (3)°. The mean planes of the ethyl acetate group [C1/C2/O1/O2/C8/C9; maximum deviation of 0.051 (2) Å for atom O2] and the dithi­azane ring (S1/S2/C1–C4) in compound (I) are inclined to one another by 17.56 (13)°. The corresponding dihedral angle in compound (II) is 11.60 (12)°. In the crystals, the mol­ecules stack along [100] in (I) and [010] in (II), and there are no significant inter­molecular inter­actions present in either.

## Synthesis and crystallization   

The title compound was prepared according to a method proposed by Thuillier & Vialle (1962 ▸). Potassium carbonate, K_2_CO_3_, (42 g, 0.3 mol) and the corresponding active methyl­ene compound, ethyl 2-cyano­acetate, (0.1 mol) were taken in 50 ml of DMF. The reaction mixture was stirred magnetically, then carbon di­sulfide (9 ml, 0.15 mol) was added at all once at room temperature. The stirring was maintained for 10 min before the dropwise addition of 1,3-di­bromo­propane (0.12 mol) over a period of 20 min. After stirring at room temperature for 7 h, ice-cold water (500 ml) was added to the reaction mixture. The yellow precipitate that formed was filtered, dried and then purified by recrystallization from ethanol (yield 93%, m.p. 368 K). The title compound exhibited the following characteristics: molar mass is *M*_w_ = 229 g mol^−1^. FT–IR (cm^−1^): 1700 (C=O), 1246–1004 [C—O (ester)], 2206 (C≡N), 1437 (C=C). ^1^H NMR (CDCl_3_, δ p.p.m., 250 MHz): 1.35 (*t*, 3H, CH_3_—CH_2_), 2.30 (*m*, 2H, CH_2_), 3.00 (*t*, 2H, CH_2_S), 3.10 (*t*, 2H, CH_2_S), 4.30 (*q*, 2H, CH_2_O). ^13^C NMR (CDCl_3_, δ p.p.m., 250 MHz):14.22 (*s*, CH_3_—CH_2_—O), 23.36 (*s*, S—CH_2_—CH_2_—CH_2_—S), 28.99 (*s*, S—CH_2_—CH_2_—CH_2_—S), 61.26 (*s*, CH_3_–CH_2_), 120.55 (*s*, CN), 76.69 (s, O=C—C=C), 165.56 (*s*, O—-C=O). MS: *m*/*z* 229.

## Refinement   

Crystal data, data collection and structure refinement details are summarized in Table 1 ▸. The H atoms were included in calculated positions and treated as riding atoms: C—H = 0.96–0.97 Å with *U*_iso_(H) = 1.5*U*_eq_(C-meth­yl) and 1.2*U*_eq_(C) for other H-atoms.

## Supplementary Material

Crystal structure: contains datablock(s) I, global. DOI: 10.1107/S2056989017017893/su5404sup1.cif

Structure factors: contains datablock(s) I. DOI: 10.1107/S2056989017017893/su5404Isup2.hkl

CCDC reference: 1811267

Additional supporting information:  crystallographic information; 3D view; checkCIF report

## Figures and Tables

**Table 1:** Experimental details

Crystal data	
Chemical formula	C_9_H_11_NO_2_S_2_
*M*_r_	229.31
Crystal system, space group	Monoclinic, *I*2/*a*
Temperature (K)	293
*a*, *b*, *c* (Å)	15.826 (3), 8.0772 (6), 18.431 (2)
β (°)	111.830 (16)
*V* (Å^3^)	2187.1 (5)
*Z*	8
Radiation type	Mo *K*α
μ (mm^−1^)	0.46
Crystal size (mm)	0.48 × 0.27 × 0.13

Data collection	
Diffractometer	Agilent Xcalibur Eos
Absorption correction	Multi-scan (*CrysAlis PRO*; Agilent, 2013 ▸)
*T*_min_, *T*_max_	0.334, 1.000
No. of measured, independent and observed [*I* > 2σ(*I*)] reflections	4539, 2132, 1667
*R*_int_	0.035
(sin θ/λ)_max_ (Å^−1^)	0.617

Refinement	
*R*[*F* ^2^ > 2σ(*F* ^2^)], *wR*(*F* ^2^), *S*	0.049, 0.138, 1.08
No. of reflections	2132
No. of parameters	127
H-atom treatment	H-atom parameters constrained
Δρ_max_, Δρ_min_ (e Å^−3^)	0.46, −0.34

Computer programs: *CrysAlis PRO* (Agilent, 2013 ▸), *SIR92* (Altomare *et al.*, 1994 ▸), *ORTEP-3 for Windows* (Farrugia, 2012 ▸) and *Mercury* (Macrae *et al.*, 2008 ▸), *SHELXL2016* (Sheldrick, 2015 ▸), *PLATON* (Spek, 2009 ▸) and *publCIF* (Westrip, 2010 ▸).

## supplementary crystallographic information

### Crystal data

**Table:**

C_9_H_11_NO_2_S_2_	*F*(000) = 960
*M**_r_* = 229.31	*D*_x_ = 1.393 Mg m^−^^3^
Monoclinic, *I*2/*a*	Mo *K*α radiation, λ = 0.71073 Å
*a* = 15.826 (3) Å	Cell parameters from 1541 reflections
*b* = 8.0772 (6) Å	θ = 3.7–28.9°
*c* = 18.431 (2) Å	µ = 0.46 mm^−^^1^
β = 111.830 (16)°	*T* = 293 K
*V* = 2187.1 (5) Å^3^	Needle, pale yellow
*Z* = 8	0.48 × 0.27 × 0.13 mm

### Data collection

**Table:**

Agilent Xcalibur Eos diffractometer	2132 independent reflections
Graphite monochromator	1667 reflections with *I* > 2σ(*I*)
Detector resolution: 8.02 pixels mm^-1^	*R*_int_ = 0.035
ω scans	θ_max_ = 26.0°, θ_min_ = 3.4°
Absorption correction: multi-scan (CrysAlis PRO; Agilent, 2013)	*h* = −17→19
*T*_min_ = 0.334, *T*_max_ = 1.000	*k* = −9→9
4539 measured reflections	*l* = −22→20

### Refinement

**Table:**

Refinement on *F*^2^	Primary atom site location: structure-invariant direct methods
Least-squares matrix: full	Secondary atom site location: difference Fourier map
*R*[*F*^2^ > 2σ(*F*^2^)] = 0.049	Hydrogen site location: inferred from neighbouring sites
*wR*(*F*^2^) = 0.138	H-atom parameters constrained
*S* = 1.08	*w* = 1/[σ^2^(*F*_o_^2^) + (0.0673*P*)^2^ + 0.4223*P*] where *P* = (*F*_o_^2^ + 2*F*_c_^2^)/3
2132 reflections	(Δ/σ)_max_ < 0.001
127 parameters	Δρ_max_ = 0.46 e Å^−^^3^
0 restraints	Δρ_min_ = −0.34 e Å^−^^3^

### Special details

**Table:**

Geometry. All esds (except the esd in the dihedral angle between two l.s. planes) are estimated using the full covariance matrix. The cell esds are taken into account individually in the estimation of esds in distances, angles and torsion angles; correlations between esds in cell parameters are only used when they are defined by crystal symmetry. An approximate (isotropic) treatment of cell esds is used for estimating esds involving l.s. planes.

### Fractional atomic coordinates and isotropic or equivalent isotropic displacement parameters (Å^2^)

**Table:**

	*x*	*y*	*z*	*U*_iso_*/*U*_eq_	
S2	0.12671 (5)	0.08363 (8)	0.29431 (4)	0.0555 (3)	
S1	0.11241 (5)	0.40461 (8)	0.37170 (4)	0.0589 (3)	
O1	0.14349 (14)	−0.1692 (2)	0.40827 (11)	0.0641 (5)	
O2	0.12483 (13)	−0.1107 (2)	0.52062 (11)	0.0592 (5)	
N1	0.1307 (2)	0.2907 (3)	0.55919 (14)	0.0741 (7)	
C1	0.13376 (16)	−0.0706 (3)	0.45311 (14)	0.0477 (6)	
C2	0.13016 (16)	0.1100 (3)	0.44342 (13)	0.0447 (6)	
C3	0.13080 (18)	0.2096 (3)	0.50818 (15)	0.0512 (6)	
C4	0.12494 (15)	0.1895 (3)	0.37572 (14)	0.0449 (6)	
C5	0.1624 (2)	0.4577 (4)	0.30133 (16)	0.0620 (7)	
H5A	0.166410	0.577323	0.299200	0.074*	
H5B	0.224000	0.414405	0.319553	0.074*	
C6	0.1113 (2)	0.3938 (3)	0.21943 (16)	0.0639 (7)	
H6A	0.153558	0.381976	0.192819	0.077*	
H6B	0.065809	0.474754	0.190915	0.077*	
C7	0.06477 (19)	0.2289 (3)	0.21774 (15)	0.0615 (7)	
H7A	0.053615	0.176859	0.167585	0.074*	
H7B	0.006049	0.249883	0.221227	0.074*	
C8	0.1298 (2)	−0.2859 (3)	0.54028 (17)	0.0617 (7)	
H8A	0.185483	−0.334042	0.539292	0.074*	
H8B	0.078396	−0.344992	0.503306	0.074*	
C9	0.1279 (2)	−0.2958 (4)	0.62053 (19)	0.0731 (9)	
H9A	0.131070	−0.409675	0.636284	0.110*	
H9B	0.179046	−0.236653	0.656364	0.110*	
H9C	0.072531	−0.247544	0.620570	0.110*	

### Atomic displacement parameters (Å^2^)

**Table:**

	*U*^11^	*U*^22^	*U*^33^	*U*^12^	*U*^13^	*U*^23^
S2	0.0705 (5)	0.0497 (4)	0.0437 (4)	0.0033 (3)	0.0182 (3)	−0.0037 (3)
S1	0.0862 (5)	0.0418 (4)	0.0494 (4)	0.0049 (3)	0.0259 (4)	0.0024 (3)
O1	0.0886 (14)	0.0476 (10)	0.0518 (11)	0.0075 (9)	0.0211 (10)	−0.0022 (9)
O2	0.0821 (13)	0.0399 (9)	0.0561 (11)	0.0057 (8)	0.0262 (10)	0.0067 (8)
N1	0.115 (2)	0.0547 (14)	0.0513 (14)	0.0002 (13)	0.0293 (15)	−0.0027 (12)
C1	0.0463 (13)	0.0469 (13)	0.0407 (13)	0.0027 (10)	0.0057 (10)	0.0025 (11)
C2	0.0471 (12)	0.0443 (13)	0.0349 (11)	0.0026 (10)	0.0064 (10)	−0.0013 (10)
C3	0.0616 (15)	0.0447 (13)	0.0413 (13)	0.0019 (11)	0.0122 (12)	0.0057 (11)
C4	0.0417 (12)	0.0435 (13)	0.0422 (13)	0.0018 (9)	0.0073 (10)	0.0005 (10)
C5	0.0682 (17)	0.0556 (15)	0.0593 (17)	−0.0084 (13)	0.0203 (14)	0.0065 (13)
C6	0.080 (2)	0.0629 (17)	0.0485 (15)	−0.0046 (14)	0.0235 (14)	0.0030 (14)
C7	0.0710 (17)	0.0669 (17)	0.0389 (13)	−0.0043 (14)	0.0116 (13)	0.0008 (13)
C8	0.0763 (19)	0.0408 (13)	0.0678 (19)	0.0036 (12)	0.0266 (15)	0.0098 (12)
C9	0.098 (2)	0.0544 (17)	0.078 (2)	0.0146 (15)	0.0454 (19)	0.0181 (15)

### Geometric parameters (Å, º)

**Table:**

S2—C4	1.736 (2)	C5—H5B	0.9700
S2—C7	1.817 (3)	C6—C7	1.517 (4)
S1—C4	1.747 (2)	C6—H6A	0.9700
S1—C5	1.805 (3)	C6—H6B	0.9700
O1—C1	1.198 (3)	C7—H7A	0.9700
O2—C1	1.343 (3)	C7—H7B	0.9700
O2—C8	1.456 (3)	C8—C9	1.492 (4)
N1—C3	1.146 (3)	C8—H8A	0.9700
C1—C2	1.469 (3)	C8—H8B	0.9700
C2—C4	1.378 (3)	C9—H9A	0.9600
C2—C3	1.436 (3)	C9—H9B	0.9600
C5—C6	1.514 (4)	C9—H9C	0.9600
C5—H5A	0.9700		
			
C4—S2—C7	100.12 (13)	C5—C6—H6B	108.9
C4—S1—C5	101.16 (13)	C7—C6—H6B	108.9
C1—O2—C8	116.8 (2)	H6A—C6—H6B	107.7
O1—C1—O2	124.3 (2)	C6—C7—S2	115.69 (19)
O1—C1—C2	125.9 (2)	C6—C7—H7A	108.4
O2—C1—C2	109.8 (2)	S2—C7—H7A	108.4
C4—C2—C3	118.1 (2)	C6—C7—H7B	108.4
C4—C2—C1	124.0 (2)	S2—C7—H7B	108.4
C3—C2—C1	117.9 (2)	H7A—C7—H7B	107.4
N1—C3—C2	179.1 (3)	O2—C8—C9	106.2 (2)
C2—C4—S2	122.55 (18)	O2—C8—H8A	110.5
C2—C4—S1	117.99 (18)	C9—C8—H8A	110.5
S2—C4—S1	119.43 (14)	O2—C8—H8B	110.5
C6—C5—S1	114.9 (2)	C9—C8—H8B	110.5
C6—C5—H5A	108.5	H8A—C8—H8B	108.7
S1—C5—H5A	108.5	C8—C9—H9A	109.5
C6—C5—H5B	108.5	C8—C9—H9B	109.5
S1—C5—H5B	108.5	H9A—C9—H9B	109.5
H5A—C5—H5B	107.5	C8—C9—H9C	109.5
C5—C6—C7	113.3 (2)	H9A—C9—H9C	109.5
C5—C6—H6A	108.9	H9B—C9—H9C	109.5
C7—C6—H6A	108.9		
			
C8—O2—C1—O1	1.6 (4)	C7—S2—C4—C2	153.6 (2)
C8—O2—C1—C2	−178.2 (2)	C7—S2—C4—S1	−24.31 (17)
O1—C1—C2—C4	9.4 (4)	C5—S1—C4—C2	153.59 (19)
O2—C1—C2—C4	−170.8 (2)	C5—S1—C4—S2	−28.43 (18)
O1—C1—C2—C3	−171.5 (2)	C4—S1—C5—C6	65.6 (2)
O2—C1—C2—C3	8.2 (3)	S1—C5—C6—C7	−32.9 (3)
C3—C2—C4—S2	178.22 (18)	C5—C6—C7—S2	−37.1 (3)
C1—C2—C4—S2	−2.7 (3)	C4—S2—C7—C6	65.9 (2)
C3—C2—C4—S1	−3.9 (3)	C1—O2—C8—C9	174.2 (2)
C1—C2—C4—S1	175.18 (18)		
